# Genetic Epidemiology and Clinical Features of Hereditary Hearing Impairment in the Taiwanese Population

**DOI:** 10.3390/genes10100772

**Published:** 2019-10-01

**Authors:** Chen-Chi Wu, Cheng-Yu Tsai, Yi-Hsin Lin, Pey-Yu Chen, Pei-Hsuan Lin, Yen-Fu Cheng, Che-Ming Wu, Yin-Hung Lin, Chee-Yee Lee, Jargalkhuu Erdenechuluun, Tien-Chen Liu, Pei-Lung Chen, Chuan-Jen Hsu

**Affiliations:** 1 Department of Otolaryngology, National Taiwan University Hospital, Taipei 10002, Taiwancjhsu@ntu.edu.tw (C.-J.H.); 2 Graduate Institute of Medical Genomics and Proteomics, National Taiwan University College of Medicine, Taipei 10055, Taiwan; 3 Department of Otolaryngology, Mackay Memorial Hospital, Taipei 10449, Taiwan; 4 Department of Otolaryngology, National Taiwan University Hospital Yunlin Branch, Yunlin 64041, Taiwan; 5 Department of Medical Research, Taipei Veterans General Hospital, Taipei 11217, Taiwan; 6 Department of Otolaryngology-Head and Neck Surgery, Taipei Veterans General Hospital, Taipei 11217, Taiwan; 7 Department of Otolaryngology-Head and Neck Surgery, Chang Gung Memorial Hospital, Chang Gung University, Linkou 33302, Taiwan; 8 Department of Otolaryngology, Buddhist Tzuchi General Hospital, Taichung Branch, Taichung 42743, Taiwan; 9 Department of Otolaryngology, Mongolian National University of Medical Sciences, Ulaanbaatar 14210, Mongolia; 10 The EMJJ Otolaryngology Hospital, Ulaanbaatar 14210, Mongolia; 11 Department of Otolaryngology, National Center for Maternal and Child Health, Ulaanbaatar 16060, Mongolia; 12 Department of Medical Genetics, National Taiwan University Hospital, Taipei 10041, Taiwan

**Keywords:** next-generation sequencing, population genomics, deafness, genetic diagnosis, genetic examination, precision medicine

## Abstract

Hereditary hearing impairment (HHI) is a common but heterogeneous clinical entity caused by mutations in a plethora of deafness genes. Research over the past few decades has shown that the genetic epidemiology of HHI varies significantly across populations. In this study, we used different genetic examination strategies to address the genetic causes of HHI in a large Taiwanese cohort composed of >5000 hearing-impaired families. We also analyzed the clinical features associated with specific genetic mutations. Our results demonstrated that next-generation sequencing-based examination strategies could achieve genetic diagnosis in approximately half of the families. Common deafness-associated genes in the Taiwanese patients assessed, in the order of prevalence, included *GJB2*, *SLC26A4*, *OTOF*, *MYO15A*, and *MTRNR1*, which were similar to those found in other populations. However, the Taiwanese patients had some unique mutations in these genes. These findings may have important clinical implications for refining molecular diagnostics, facilitating genetic counseling, and enabling precision medicine for the management of HHI.

## 1. Introduction

Hearing impairment is the most common inherited sensory defect. It is estimated that permanent sensorineural hearing impairment (SNHI) occurs in approximately 1.9 out of 1000 live births [1], and including late-onset SNHI, the disorder may affect 2% of school-age children [2,3]. About one-half to two-thirds of SNHI cases in children are attributed to genetic causes and are classified as hereditary hearing impairment (HHI) [4]. To date, more than 100 genes have been identified as causally related to HHI (http://hereditaryhearingloss.org). Because different genetic mutations represent different pathogeneses and are related to different clinical outcomes, identifying etiology is crucial in the assessment and management of childhood SNHI.

Over the past two decades, research has revealed that the genetic epidemiology of HHI varies across populations. For instance, c.235delC, c.35delG, and c.167delT are the most common *GJB2* mutations in East Asians [5,6,7], Caucasians [8,9,10,11], and Ashkenazi Jews [12], respectively, whereas the c.-23+1G>A mutation is unique to Southwest and South Asians [13,14,15,16]. Similarly, predominant *SLC26A4* mutations differ among populations; p.T416P and c.1001G>A are the most predominant mutations in Caucasians [17,18], p.H723R in Japanese [19] and Koreans [20], and c.919-2A>G in Han Chinese [21]. These findings underscore the importance of collecting regional data when genetic examination for SNHI is performed in a specific population.

Taiwan is an island in the Pacific Ocean with a population of 23.4 million people. As an immigrant population, approximately 98% of Taiwanese are of Han Chinese ancestry, whereas the remaining 2% are of aboriginal ancestry (Austronesian) [22]. According to a recent population structure study based on a nationwide biobank, the Taiwanese Han Chinese were clustered into three groups: 5% were of northern Han Chinese ancestry, 79.9% were of southern Han Chinese ancestry, and 14.5% belonged to a third group that is genetically distinct from neighboring Southeast Asians and Austronesian tribes but similar to other southern Han Chinese [23]. As such, the predominant majority of the Taiwanese identify themselves as Han Chinese.

To address the genetic epidemiology of HHI in the Taiwanese population, we have established a large SNHI cohort composed of >5000 families in Taiwan over the past 15 years. In addition to achieving genetic diagnoses for HHI in these SNHI families, our results also demonstrated certain translational applications of genetic examination for HHI in clinical practice [24,25]. In this article, we analyzed the genetic profiles in this large Taiwanese cohort and reported the clinical features associated with specific genetic mutations

## 2. Materials and Methods 

### 2.1. Subject Recruitment and Phenotype Characterization

From 2005 to 2019, a total of 5314 unrelated families were enrolled in the SNHI cohort of the Department of Otolaryngology, National Taiwan University Hospital. At least one member of each family suffered from idiopathic SNHI. Patients were excluded if they (1) were more than 40 years old, (2) had conductive or mixed-type hearing impairment, (3) had a history of perinatal insults, such as prematurity or kernicterus, (4) had previous noise or ototoxic agents exposure, or (5) had no complete records of their medical history available. All participants in this study identified themselves as Han Chinese. There were 198 families with multiple affected members, including 62 families compatible with autosomal dominant inheritance, 123 families compatible with autosomal recessive inheritance, and 13 families compatible with X-linked inheritance. The probands of most families had non-syndromic SNHI; however, some probands revealed clinical features compatible with specific syndromes, such as branchio-oto-renal syndrome (*n* = 15), Waardenburg syndrome (*n* = 13), Alport syndrome (*n* = 9), and Usher syndrome (*n* = 10).

For the affected member(s) of each family, a comprehensive family history, personal medical history, physical examination, audiological results, and imaging results (when indicated) were ascertained. The audiological results were evaluated with pure tone audiograms, otoacoustic emissions, auditory brainstem response, or auditory steady-state response, depending on age or neurological status [26]. Imaging results were obtained using high-resolution computed tomography and/or magnetic resonance imaging; abnormalities of the inner ear and cochlear nerve were determined according to criteria in the literature [27,28,29]. Informed consent were provided by all subjects and/or their parents prior to the genetic testing, and all procedures were approved by the Research Ethics Committees of the National Taiwan University Hospital.

All subjects gave their informed consent for inclusion before they participated in the study. The study was conducted in accordance with the Declaration of Helsinki, and the protocol was approved by the Ethics Committee of 201712138RINA (Project identification code).

### 2.2. Conventional Genetic Examination Using Sanger Sequencing

Since 2005, we have standardized a genetic examination protocol for mutation screening for three common deafness genes: *GJB2*, *SLC26A4*, and *MTRNR1* [30,31,32]. Sanger sequencing was performed on exon 2 of *GJB2*, all 21 exons of *SLC26A4*, and the mitochondrial 12S rRNA gene (*MTRNR1*).

### 2.3. Next-Generation Sequence (SGS)-Based Genetic Examination

Since 2013, we have started to develop diagnostic panels using next-generation sequencing (NGS) technology [33]. We have been upgrading our NGS-based diagnostic panels continuously: the initial version included 80 deafness genes, which has gradually been expanded to 213 genes in our current version. The NGS-based diagnostic panel was originally designed to address families unresolved by conventional genetic examinations, but has been commercialized and utilized as a first-tier diagnostic platform since 2018.

For NGS-based genetic examinations, sample preparation, DNA sequencing, and data analyses were performed as previously described [33,34,35,36]. Briefly, paired-end reads were aligned, sorted, and converted by BWA-MEM and Picard. Variants, including single nucleotide substitution and small deletions/insertions, were called by GATK HaplotypeCaller. ANNOVAR was used to annotate pathogenicity prediction by PolyPhen-2 (HumVar), SIFT, LRT, MutationTaster, MutationAssessor, FATHMM, and MetaLR. Allele frequencies in the 1000 Genomes Project (http://www.1000genomes.org/), the Exome Aggregation Consortium projects (ExAC) (http://exac.broadinstitute.org/), and the Genome Aggregation Database (gnomAD) (http://gnomad.broadinstitute.org/) were also annotated by ANNOVAR.

After variant annotation, we identified previously reported variants through the Deafness Variation Database (http://deafnessvariationdatabase.org/, last accessed 9 January, 2019) and ClinVar (https://www.ncbi.nlm.nih.gov/clinvar/, last accessed 9 June, 2019). All variants, including reported and unreported variants, were categorized according to the American College of Medical Genetics and Genomics (ACMG) guidelines. Sanger sequencing was performed to confirm the identified variants and examine co-segregation of the genotype and SNHI phenotype among the family members. Variants fitting the criteria of “pathogenic” or “likely pathogenic” were reported as disease-causing. Genetic diagnosis was considered confirmed when either one disease-causing variant was detected in dominant genes or bi-allelic disease-causing variants were detected in recessive genes. 

## 3. Results

### 3.1. Diagnostic Yields of the Genetic Examinations

**Figure 1:**
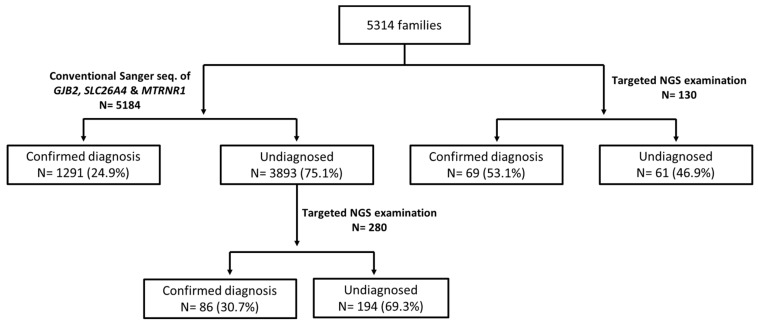
Summary of the genetic results in the 5314 Taiwanese families included in this study. The probands of 5184 families received conventional Sanger sequencing of three common deafness genes (i.e., *GJB2*, *SLC26A4*, and *MTRNR1)*, and 1291 (24.9%) of them had confirmed diagnosis. Among the remaining 3893 undiagnosed probands, 280 of them further received a second-phase targeted NGS examination, and 86 (30.7%) had confirmed diagnosis. On the other hand, 130 families directly received targeted NGS examination without screening of common deafness gene *a priori*. Of them, 69 (53.1%) had confirmed diagnosis. NGS, next-generation sequencing.

Among the probands of 5314 families, 5184 were subjected to conventional genetic examination of the three common deafness genes (Figure 1). Confirmative genetic diagnosis was achieved in 1291 (24.9%) of the 5184 probands, including 895 (17.3%) with bi-allelic *GJB2* mutations, 294 (5.7%) with bi-allelic *SLC26A4* mutations, and 102 (2.0%) with the m.1555A>G mutation. Of the 3893 probands without confirmed diagnosis, 280 underwent targeted NGS examination as the second phase screening, and 86 (30.7%) of them were confirmed to have disease-causing variants in other deafness genes, as seen in Table 1.

From 2018 to 2019, the probands of 130 SNHI families directly received targeted NGS examination instead of conventional genetic examination. Definite genetic diagnosis was achieved in 69 (53.1%) of the 130 probands, as seen in Table 1. Common genetic causes included mutations in *GJB2* (*n* = 42, 32.3%), *MYO15A* (*n* = 8, 6.2%), *SLC26A4* (*n* = 5, 3.8%), and *OTOF* (*n* = 4, 3.1%). 

**Figure 2:**
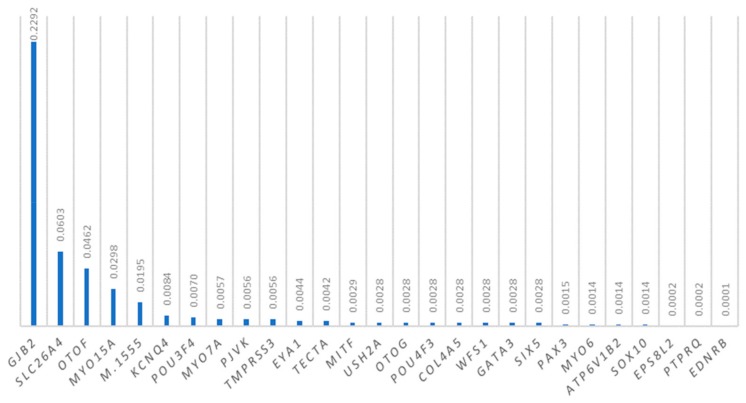
Allele frequencies of deafness-associated genetic mutations in the Taiwanese families.

The allele frequencies of mutations in deafness genes in the Taiwanese families are shown in Figure 2. The most prevalent pathogenic variants included *GJB2* mutations (22.92%), *SLC26A4* mutations (6.03%), *OTOF* mutations (4.62%), *MYO15A* mutations (2.98%), and the m.1555A>G mutation (1.95%).

### 3.2. GJB2 Mutations

In our cohort, the probands of 935 families were diagnosed to have bi-allelic recessive *GJB2* mutations, and two probands were diagnosed to have mono-allelic dominant *GJB2* mutations. The most common *GJB2* mutations in the Taiwanese SNHI probands included p.V37I (85.9%), c.235delC (11.5%), and c.299_300delAT (1.7%). The *GJB2* mutations identified in the 937 Taiwanese families are shown in Table 2.

Different *GJB2* genotypes were associated with different hearing severity and audiogram shapes. Patients who were homozygous or compound heterozygous for p.V37I showed milder SNHI than those with other *GJB2* mutations. Nonetheless, progressive SNHI could occur in patients with different *GJB2* genotypes with an average progression rate of ~0.55 dBHL per year. Notably, the progression rate did not differ significantly among patients with different *GJB2* genotypes [37].

### 3.3. SLC26A4 Mutations

Clinically, *SLC26A4* mutations contribute to non-syndromic enlarged vestibular aqueduct (DFNB4, MIM 600791) and Pendred syndrome (PS, MIM 274600). To date, we have performed comprehensive genetic screening for *SLC26A4* in the probands of 361 families with DFNB4 or PS. In total, *SLC26A4* mutations were detected in 346 probands, including 300 (83.1%) with bi-allelic and 46 (12.7%) with mono-allelic *SLC26A4* mutations. The *SLC26A4* mutations identified in the 346 Taiwanese families are shown in Table 3. We did not observe differences in clinical features, including radiological findings, the presence of goiters, and audiological results, in our patients with different *SLC26A4* genotypes [31,38].

### 3.4. OTOF Mutations

To date, we have performed comprehensive genetic examination in the probands of 54 families with non-acquired auditory neuropathy. The *OTOF* mutations identified in these families are shown in Table 4. A total of 22 probands were diagnosed with bi-allelic *OTOF* mutations, including eight homozygous for p.E1700Q and 14 compound heterozygous for p.E1700Q and another variant. Eight probands were found to have mono-allelic *OTOF* mutations. It is unclear whether auditory neuropathy in these mono-allelic probands was caused by the presence of another hidden *OTOF* mutation or by other etiologies. 

In our cohort, all patients with bi-allelic *OTOF* mutations presented phenotypes compatible with auditory neuropathy of autosomal recessive inheritance. The hearing thresholds on behavioral audiometries ranged from moderate to profound SNHI, with two-thirds of them showing moderate SNHI and one-third showing severe-to-profound SNHI at initial presentation. Most of the patients (75%) failed newborn hearing screening at birth. In the twelve patients with longitudinal hearing data available for analyses, seven (58.3%) showed progressive hearing loss, four (33.3%) showed stable hearing loss, and one (8.3%) demonstrated fluctuation in the hearing levels. 

### 3.5. MYO15A Mutations

Recessive *MYO15A* mutations are the fourth most common genetic cause of SNHI in Taiwanese patients. In our cohort, a total of 22 families (27 patients) were diagnosed to have bi-allelic pathogenic or likely pathogenic variants in *MYO15A.* The *MYO15A* mutations identified in these Taiwanese patients are shown in Table 5. The most prevalent variants were c.3524dupA (20.5%, 9/44), c.8182C>G (9.1%, 4/44), and c.3757-32_3757-1del (6.8%, 3/44). Several variants are unique to the Taiwanese patients and have never been identified in other populations, including c.3757-32_3757-1del, c.4101C>A, c.4457G >T, c.4760T>C, c.4761_4762insGTTTCTAT, c.5443C>A, c.6956+1G>A, c.8182C >G, c.8602-1G>C, and c.9408G>C.

In our cohort, more than half (77.8%, 21/27) of the patients with bi-allelic *MYO15A* mutations presented with severe to profound SNHI. Patients with mutations in the N-terminal domain showed less severe SNHI and more low-tone residual hearing compared to those with mutations in the non-N terminal domain (unpublished data). Approximately one-third of our patients showed progression of SNHI during the follow-up period. None of them developed vestibular symptoms. 

### 3.6. MTRNR1 Mutations

The m.1555A>G mutation in the *MTRNR1* gene accounted for ~2% of Taiwanese families (~10% of multiplex families) with idiopathic SNHI [39]. The m.1494C>T mutation that has been reported as pathogenic in other populations [40,41] was not detected in our cohort. In Taiwanese patients, m.1555A>G was identified in a variety of mtDNA backgrounds (i.e., the mitochondrial haplogroups), suggesting that this mutation arose from multiple origins in the Taiwanese population [39]. Interestingly, families with various haplogroups demonstrated different penetrance of SNHI, indicating that the mtDNA background might exert effects on the disease expression [39].

### 3.7. Mutations in Other Deafness Genes

In our cohort, pathogenic variants were also detected in a number of other deafness genes, including *KCNQ4*, *POU3F4*, *MYO7A*, *TMPRSS3*, *EYA1*, *TECTA*, *MITF*, *POU4F3*, *PJVK*, *COL4A5*, *WFS1*, *GATA3*, *SIX5*, *USH2A*, *OTOG*, *PAX3*, *MYO6*, *ATP6V1B2*, *SOX10*, *PTPRQ*, *EPS8L2*, and *EDNRB*, as shown in Table 6. Some variants are unique to Taiwanese patients and have never been identified in other populations.

Of these, mutations in 15 genes, including *KCNQ4*, *MYO7A*, *EYA1*, *TECTA*, *MITF*, *POU4F3*, *WFS1*, *GATA3*, *SIX5*, *PAX3*, *MYO6*, *ATP6V1B2*, *SOX10*, *EDNRB*, and *PTPRQ* are associated with autosomal dominant inheritance; mutations in 12 genes are associated with syndromic hearing loss, including Waardenberg syndrome (*MITF*, *PAX3*, and *SOX10*), BOR syndrome (*SIX5* and *EYA1*), hypoparathyroidism, sensorineural deafness, and renal dysplasia (HDR) syndrome (*GATA3*), Wolfram syndrome (*WFS1*), Usher syndrome (*USH2A* and *MYO7A*), Pendred syndrome (*SLC26A4*), Alport syndrome (*COL4A5*), congenital deafness with onychodystrophy (*ATP6V1B2*), as shown in Table 1.

## 4. Discussion

### 4.1. Comparison of Diagnostic Yields among Different Examination Strategies

Genetic diagnosis could be confirmed in approximately 25% of the SNHI families by conventional Sanger sequencing of the three common deafness genes. By contrast, the diagnosis rate increased to approximately 50% using the targeted NGS examination strategy. The diagnostic yield of the targeted NGS genetic examination is approaching the reported ratio (i.e., one-half to two-thirds) of HHI in pediatric SNHI [4]. To date, several NGS-based diagnostic panels for HHI, including ours, have been designed [4,45,46,47,48,49]. There is solid evidence that the use of the NGS technique can significantly increase the diagnostic yield of genetic examination for HHI and facilitate the genetic counseling for the affected families.

### 4.2. GJB2 Mutations

*GJB2* mutations are the most common genetic cause of SNHI in Taiwanese families, as in many other populations [37]. To date, more than 340 pathogenic recessive or dominant *GJB2* mutations have been reported (http://deafnessvariationdatabase.org/) [38]. The prevalence of *GJB2* mutations in hearing-impaired patients varied significantly across different populations, ranging from 5.6% in the sub-Saharan Africa population to 27.1% in the European population [37]. The mutation spectra also differed among different ethnic groups.

Notably, the p.V37I variant was exceedingly prevalent not only in hearing-impaired patients but also in the normal Taiwanese population. According to our population-based genetic screenings in newborns, the allele frequency of p.V37I is estimated to be 8.5% in the general Taiwanese population [50,51]. A high prevalence of the p.V37I variant has also been reported in patients recruited from south China [52] and Thailand [53].

Interestingly, our recent collaboration with Erdenechuluun et al. in Mongolia revealed a unique *GJB2* mutation spectrum in Mongolian patients with SNHI [54]. Three *GJB2* mutations that are prevalent in other populations, including c.235delC in East Asians [5,6,7], c.35delG in Caucasians [8,9,10], and c.-23+1G>A in Southwest and South Asians [13,14,15,16], were simultaneously detected in Mongolian patients. Haplotype analyses further confirmed founder effects for each of the three mutations, indicating that each mutation was independently derived from its ancestral origin.

The severity of *GJB2*-associated SNHI varies from late-onset mild hearing loss to congenital, severe-to-profound deafness, and is highly dependent on the genotypes. Patients with two “truncating” mutation alleles (such as c.235delC) usually exhibit severe-to-profound SNHI, whereas those with at least one “non-truncating” mutation allele (such as p.V37I) are associated with a milder phenotype [55,56,57,58]. Diversity in auditory phenotypes among patients with the same *GJB2* genotype was also reported [37,55,59]. In our recent study, we used generalized estimating equation (GEE) analyses to establish a predictive model for SNHI in patients with *GJB2* mutations [37]. This predictive model for SNHI may have important clinical implications in guiding follow-up protocols and designing treatment plans in patients with *GJB2* mutations.

### 4.3. SLC26A4 Mutations

Recessive *SLC26A4* mutations are the second most common genetic cause of SNHI in Taiwanese patients [30]. More than 400 pathogenic variants of *SLC26A4* have been reported (http://deafnessvariationdatabase.org/) [60]. The mutation spectra of *SLC26A4* differ significantly among different ethnic backgrounds [17,19,61,62]. In Taiwanese patients, the most prevalent *SLC26A4* mutation is c.919-2A>G [31], followed by the p.H723R mutation, which is the most common *SLC26A4* mutation in Japanese [61,63] and Korean [61,62] patients. Haplotype analyses confirmed the founder effect of c.919-2A>G in Taiwanese patients [31]. 

*SLC26A4* mutations are distributed over all 21 exons of the gene and are mostly single nucleotide variations, yet copy number variations were occasionally reported [36,64,65,66]. As such, genetic examination using conventional sequencing strategies is highly laborious and may fail to detect the pathogenic variants. On the contrary, our recent study demonstrated the utility of a NGS-based panel in addressing *SLC26A4*-related SNHI by identifying various types of mutations with satisfactory diagnostic yields in one step [36].

Approximately 83% of the DFNB4/PS patients in our cohort were confirmed to have bi-allelic *SLC26A4* mutations, which was comparable to that observed in Japanese (47–66%) [19,67], Korean (81%) [20], and Chinese (65–88%) [68,69] patients, and significantly higher than that observed in Caucasian patients (16–24%) [70,71]. Although positive genotype-phenotype correlations have been documented in some series [67,71,72], no differences in clinical features were observed in our patients with different *SLC26A4* genotypes [31,38]. Specifically, patients with mono-allelic *SLC26A4* mutations demonstrated clinical features undistinguishable from those with bi-allelic *SLC26A4* mutations, implying that there might be an undetected mutation in the no-mutation-detected *SLC26A4* allele in these heterozygotes [38].

### 4.4. OTOF Mutations

*OTOF* mutations are another common cause of childhood SNHI, and the leading genetic cause of auditory neuropathy in Taiwanese patients, as in other populations [73,74]. p.E1700Q is the predominant *OTOF* variant in Taiwanese families, accounting for approximately 70% of the total variants detected. This variant is specific in Taiwanese, and has not been reported in other populations, except in a Chinese series that reported a patient with heterozygous p.E1700Q [75]. In our previous study, we confirmed the founder effect of p.E1700Q in the Taiwanese population by haplotype analyses [76]. p.E1700Q is presumed to be disease-causing or disease-related because it is evolutionarily conserved among mammalian species and its pathogenicity is supported by multiple prediction programs (PolyPhen-2, MutationTaster, and MutationAssessor). However, we recently identified another *OTOF* variant that is in linkage disequilibrium with p.E1700Q and also likely to be pathogenic. Functional studies are underway to examine the pathogenicity of p.E1700Q.

### 4.5. MYO15A Mutations

*MYO15A* is a large gene composed of 67 exons; more than 190 *MYO15A* mutations have been documented to date [77]. The large size of the gene and the heterogeneity of the mutations have precluded the inclusion of *MYO15A* screening into routine deafness genetic examinations using conventional sequencing strategies [78,79,80]. However, recent advances in NGS technology have facilitated mutation detection and confirmed *MYO15A* mutations as a common cause of autosomal recessive non-syndromic hearing loss in several populations [81,82,83].

The phenotypes of *MYO15A* mutations vary from pre-lingual bilateral severe-to-profound SNHI [81,84] to post-lingual progressive SNHI [79,85], with residual hearing at lower frequencies occasionally noted [79,81,83,86]. In our cohort, approximately 80% of the patients with bi-allelic *MYO15A* mutations had severe to profound SNHI. We also found that patients with N-terminal domain mutations and those with non-N terminal domain mutations presented different hearing profiles, which was consistent with previous reports [86,87,88].

### 4.6. MTRNR1 Mutations

Approximate 2% of Taiwanese families with idiopathic SNHI were confirmed to have m.1555A>G mutation in the *MTRNR1* gene [39]. Large-scale genetic screenings in newborns revealed that m.1555A>G has a prevalence of approximately 1/1000 in the general Taiwanese population [50,51,89], which is comparable to that in other populations [90,91,92,93]. Notably, all participating babies in our studies with m.1555A>G passed newborn hearing screening at birth [50,51,89]. The identification of this mutation might thus have important clinical implications for these babies and their maternal relatives. Specifically, the use of aminoglycoside antibiotics should be avoided in these babies and their maternal relatives, as permanent profound SNHI might occur immediately after exposure to this medication [94,95].

### 4.7. Mutations in Other Deafness Genes

As most of the currently-available NGS diagnostic panels also include syndromic HHI genes, occasionally earlier diagnosis and earlier treatment could be facilitated in families with certain types of syndromic HHI. For instance, we previously identified a *GATA3* c.149delT mutation using our NGS panel in a family which was originally considered to have autosomal dominant non-syndromic HHI. On the basis of the genetic finding, we could later ascertain the phenotypes of HDR syndrome in this family [34]. In other words, the genetic diagnosis of HDR syndrome was confirmed prior to clinical diagnosis in this family. This is important for this family, as a close follow-up of the parathyroid and renal functions is warranted for the affected family members.

### 4.8. Strengths and Limitations of the Study

The major strength of this study lies in the demonstration of the genetic epidemiology of HHI in a large Taiwanese cohort, in which the phenotypes and genotypes of the participants were ascertained at a single institute. However, some limitations of this study merit further discussion. First, although genetic diagnosis was confirmed in approximately half of our families, it is possible that some pathogenic variants, including structural variants, non-coding variants, and mosaic variants, have been missed due to current technical bottlenecks. Second, as targeted NGS examination was not available until the last few years of the study period, only a limited number of families in our longitudinal cohort had access to it. This might have led to selection or recruitment biases.

## 5. Conclusions

In conclusion, we presented comprehensive genetic results for a large Taiwanese cohort with idiopathic SNHI and demonstrated that NGS–based or NGS–assisted genetic examination strategies could achieve diagnosis in approximately half of the families. Common deafness-associated genes in Taiwanese families, in order of prevalence, included *GJB2*, *SLC26A4*, *OTOF*, *MYO15A*, and *MTRNR1*, which are similar to those found in other populations. However, Taiwanese patients possessed some unique mutations within these common genes. These findings may have important clinical implications for refining molecular diagnostics, facilitating genetic counseling, and enabling precision medicine for the management of HHI.

## Figures and Tables

**Table 1:** Deafness genes detected with targeted NGS examination in Taiwanese patients.

Gene	No. of Patients	Inheritance Pattern	Associated Syndrome in Ref	Associated Syndrome in our Patients
86 probands with confirmed diagnosis in the second-phase targeted NGS screening				
*OTOF*	22	AR	-	-
*MYO15A*	18	AR	-	-
*KCNQ4*	5	AD	-	-
*POU3F4*	4	X-linked	-	-
*MYO7A*	5	AD/AR	Usher syndrome 1B	Non-syndromic
*EYA1*	7	AD	Branchio-oto-renal syndrome	Branchio-oto-renal syndrome
*TECTA*	3	AD/AR	-	-
*MITF*	2	AD	Waardenburg syndrome	Waardenburg syndromic
*POU4F3*	2	AD	-	-
*PJVK*	1	AR	-	-
*COL4A5*	2	X-linked	Alport syndrome	Alport syndrome
*WFS1*	2	AD/AR	Wolfram syndrome	Non-syndromic
*GATA3*	2	AD	HDR syndrome	Non-syndromic
*SIX5*	1	AD	Branchio-oto-renal syndrome	Branchio-oto-renal syndrome
*TMPRSS3*	2	AR	-	-
*PAX3*	1	AD	Waardenburg syndrome	Waardenburg syndrome
*USH2A*	2	AR	Usher syndrome 2A	-
*MYO6*	2	AD/AR	-	-
*OTOG*	1	AR	-	-
*ATP6V1B2*	1	AD	Congenital deafness with onychodystrophy	Congenital deafness with onychodystrophy
*SOX10*	1	AD	Waardenburg syndrome	Waardenburg syndrome
69 probands with confirmed diagnosis in directly targeted NGS screening				
*GJB2*	42	AR	-	-
*MYO15A*	8	AR	-	-
*SLC26A4*	5	AR	Pendred syndrome	Non-syndromic
*OTOF*	4	AR	-	
*EYA1*	2	AD	Branchio-oto-renal syndrome	Branchio-oto-renal syndrome
*KCNQ4*	1	AD	-	-
*POU3F4*	1	X-linked	-	-
*MYO7A*	1	AD/AR	Usher syndrome, type 1B	Non syndromic
*MITF*	1	AD	Waardenburg syndrome	Waardenburg syndrome
*PAX3*	1	AD	Waardenburg syndrome	Waardenburg syndrome
*EDNRB*	1	AD	-	-
*EPS8L2*	1	AR	-	-
*PTPRQ*	1	AD/AR	-	-

HDR syndrome: hypoparathyroidism, sensorineural deafness, and renal dysplasia syndrome.

**Table 2:** *GJB2* mutations detected in the Taiwanese patients.

Nucleotide Change	Amino Acid Change	Allele Count (ratio%)	Popmax AF (population) from gnomAD
Recessive mutations			
c.109G>A	p.V37I	1608 (85.9)	0.08345 (EAS)
c.235delC	p.L79Cfs *3	215 (11.5)	0.006515 (EAS)
c.299_300delAT	p.H100Rfs *14	31 (1.7)	0.0009023 (EAS)
c.427C>T	p.R143W	6 (0.3)	0.0007227 (AFR)
c.508_511dupAACG	p.A171Qfs *40	5 (0.3)	0.0003008 (EAS)
c.176_191del	p.G59Afs *18	2 (0.1)	0.0001631 (EAS)
c.95G>A	p.R32H	1 (0.1)	0.000008859 (EUP-NF)
c.107T>C	p.L36P	1 (0.1)	0.0001002 (EAS)
c.571T>C	p.F191L	1 (0.1)	0.001854 (EAS)
Dominant mutations			
c.263C>T	p.A88V	1 (0.1)	no data
c.428G>A	p.R143Q	1 (0.1)	no data
**Total**		1872 (100.0)	

NCBI Reference Sequence: NM_004004.6/NP_003995.2. *: termination; del: deletion; dup: duplication. EAS: East Asian; AFR: African; EUP-NF: European-non-Finnish.

**Table 3:** *SLC26A4* mutations detected in 346 Taiwanese families with DFNB4/PS.

Nucleotide Change	Amino Acid Change	Allele Count (ratio%)	Popmax AF (population) from gnomAD
Bi-allelic *SLC26A4* mutations			
c.919-2A >G	NA	473 (73.2)	0.005064 (EAS)
c.2168A >G	p.H723R	39 (6.0)	0.001604 (EAS)
c.1229C >T	p.T410M	17 (2.6)	0.0005879 (SA)
c.1160C >T	p.A387V	6 (0.9)	0.00005438 (EAS)
c.754T >C	p.S252P	5 (0.8)	0.00005437 (EAS)
c.1115C >T	p.A372V	5 (0.8)	No data
c.916dupG	p.V306Gfs *24	5 (0.8)	0.0001631 (EAS)
c.164+1G >C	NA	4 (0.6)	No data
c.706C >G	p.L236V	3 (0.5)	0.0002602 (LAT)
c.1343C >T	p.S448L	3 (0.5)	0.0001088 (EAS)
c.439A >G	p.M147V	2 (0.3)	0.0001087 (EAS)
c.1079C >T	p.A360V	2 (0.3)	0.000641 (EAS)
c.1173C >A	p.S391R	2 (0.3)	No data
c.1318A >T	p.K440 *	2 (0.3)	No data
c.1489G >C	p.G497R	2 (0.3)	0.00005013 (EAS)
c.2086C >T	p.Q696 *	2 (0.3)	0.0001695 (EAS)
c.2T >G	p.M1R	1 (0.2)	0.00007383 (EAS)
c.230A >T	p.K77I	1 (0.2)	No data
c.235C >T	p.R79 *	1 (0.2)	No data
c.241A >G	p.K81E	1 (0.2)	No data
c.387delC	p.F130Lfs *15	1 (0.2)	No data
c.416-1G >A	NA	1 (0.2)	0.00005437 (EAS)
c.697G >C	p.V233L	1 (0.2)	0.001353 (EAS)
c.918+2T >C	NA	1 (0.2)	0.0001387 (OTH)
c.1001+1G >A	NA	1 (0.2)	0.0003977 (EUP-NF)
c.1105A >T	p.K369 *	1 (0.2)	No data
c.1174A >T	p.N392Y	1 (0.2)	0.00005438 (EAS)
c.1226G >A	p.R409H	1 (0.2)	0.0001977 (LAT)
c.1489G >A	p.G497S	1 (0.2)	0.00005013 (EAS)
c.1520delT	p.L507 *	1 (0.2)	No data
c.1544+6T >C	NA	1 (0.2)	No data
c.1614+1G >A	NA	1 (0.2)	0.00003525 (EUP-NF)
c.1615-2A >G	NA	1 (0.2)	0.000008827 (EUP-NF)
c.1658C >A	p.P553H	1 (0.2)	0.00005438 (EAS)
c.1693_1694insA	p.C565 *	1 (0.2)	No data
c.1707+1G >A	NA	1 (0.2)	No data
c.1786C >T	p.Q596 *	1 (0.2)	No data
c.1829C >A	p.S610 *	1 (0.2)	No data
c.1975G >C	p.V659L	1 (0.2)	0.0002006 (EAS)
c.2086C >T	p.Q696T *	1 (0.2)	0.0001695 (EAS)
c.2089G >C	p.D697H	1 (0.2)	No data
c.2107C >G	p.L703V	1 (0.2)	No data
c.2162C >T	p.T721M	1 (0.2)	0.0002895 (LAT)
g.-1066C_6602Adel	NA	1 (0.2)	No data
Mono-allelic *SLC26A4* mutations			
c.919-2A >G	NA	44 (6.8)	0.005064 (EAS)
c.916_917insG	p.V306Gfs *24	1 (0.2)	0.0001631 (EAS)
c.974_977 delCTGGinsTTAAATTA	p.A325Vfs *6	1 (0.2)	No data
Total		646 (100.0)	

NCBI Reference Sequence: NM_000441.1/NP_000432.1. del: deletion; ins: insertion; dup: duplication; *: termination; fs: frameshift. EAS: East Asian; SA: South Asian; LAT: Latino; EUP-NF: European-non-Finnish; OTH: other.

**Table 4:** *OTOF* mutations detected in 30 Taiwanese families with auditory neuropathy.

Nucleotide Change	Amino Acid Change	Allele Count (ratio%)	Popmax AF (population) from gnomAD
Bi-allelic *OTOF* mutations			
c.5098G>C	p.E1700Q	30 (57.7)	0.006774 (EAS)
c.2521G>A	p.E841K	3 (5.8)	0.0002202 (EAS)
c.1498C>T	p.R500 *	1 (1.9)	0.0001387 (OTH)
c.2279T>C	p.L760P	1 (1.9)	no data
c.3704_3719del	p.D1235Afs *30	1 (1.9)	no data
c.3894+5G>C	NA	1 (1.9)	no data
c.4023+1G>A	NA	1 (1.9)	0.01178 (EAS)
c.4030C>T	p.R1344 *	1 (1.9)	no data
c.4961-1G>A	NA	1 (1.9)	no data
c.5197G>A	p.E1733K	1 (1.9)	0.00005439 (EAS)
c.5203C>T	p.R1735W	1 (1.9)	0.000008792 (EUP-NF)
c.5335C>T	p.H1779Y	1 (1.9)	no data
c.5566C>T	p.R1856W	1 (1.9)	0.0001003 (EAS)
Mono-allelic *OTOF* mutations			
c.5098G>C	p.E1700Q	6 (11.5)	0.006774 (EAS)
c.4023+1G>A	NA	1 (1.9)	0.01178 (EAS)
c.4227+5G>C	NA	1 (1.9)	0.001414 (EAS)
Total		52 (100.0)	

NCBI Reference Sequence: NM_194248.2/NP_919224.1. *: termination; fs: frameshift; del: deletion; EAS: East Asian; EUP-NF: European-non-Finnish; OTH: other.

**Table 5:** *MYO15A* mutations detected in Taiwanese patients.

Nucleotide Change	Amino Acid Change	Allele Count (ratio%)	Popmax AF (population) from gnomAD
c.3524dupA	p.Ser1176ValfsTer14	9 (20.5)	0.002152 (EAS)
c.10250_10252delCCT	p.Ser3417del	8 (18.2)	0.0004095 (EAS)
c.8182C>G	p.Arg2728Gly	4 (9.1)	0.0002226 (EAS)
c.3757-32_3757-1del	NA	3 (6.8)	0.0003583 (EAS)
c.5964+3G>A	NA	2 (4.5)	0.0003908 (EAS)
c.9408G>C	p.Trp3136Cys	2 (4.5)	0.0003583 (EAS)
c.10111C>T	p.Gln3371 *	1 (2.3)	0.00006473 (AFR)
c.10258_10260delTTC	p.Phe3420del	1 (2.3)	0.0001112 (EAS)
c.3844C>T	p.Arg1282Trp	1 (2.3)	0.0001403 (OTH)
c.4101C>A	p.Asn1367Lys	1 (2.3)	no data
c.4457G>T	p.Gly1486Val	1 (2.3)	no data
c.4642G>A	p.Ala1548Thr	1 (2.3)	0.0001239 (AFR)
c.4760T>C	p.Leu1587Pro	1 (2.3)	no data
c.4761_4762insGTTTCTAT	p.Asp1588Valfs *11	1 (2.3)	no data
c.5421_5437del	p.Glu1808Glyfs *41	1 (2.3)	no data
c.5443C>A	p.Gln1815Lys	1 (2.3)	no data
c.5977C>T	p.Arg1993Trp	1 (2.3)	0.0001712 (EAS)
c.6281G>A	p.Arg2094His	1 (2.3)	0.000008965 (EUP-NF)
c.6956+1G>A	NA	1 (2.3)	0.00006504 (EAS)
c.7708_7709insCA	p.Gln2571Hisfs * 35	1 (2.3)	0.000641 (EAS)
c.7986dupG	p.Glu2663Glyfs * 4	1 (2.3)	no data
c.8602-1G>C	NA	1 (2.3)	no data
Total		52 (100.0)	

NCBI Reference Sequence: NM_016239.4/NP_057323.3. dup: duplication; del: deletion; ins: insertion; *: termination; fs: frameshift. EAS: East Asian; AFR: African; EUP-NF: European-non-Finnish; OTH: other.

**Table 6:** Mutations of other deafness genes detected in Taiwanese patients.

Nucleotide Change	Amino Acid Change	Allele Count	Popmax AF (population) from gnomAD	References
*KCNQ4*				
c.546C>G	p.F182L	3	0.004576 (EAS)	Our cohort; Ref [42]
c.2014G>A	p.V672M	2	No data	Our cohort
c.2039C>T	p.S680F	1	0.0002006 (EAS)	Our cohort; Ref [33]
*MYO7A*				
c.689C>T	p.A230V	1	No data	Our cohort
c.1142C>T	p.T381M	1	0.0007619 (EAS)	Our cohort; Ref [33,43]
c.2557C>T	p.R853C	1	No data	Our cohort
c.6335C>G	p.S2112 *	1	No data	Our cohort
c.6470T>C	p.I2157T	1	0.00005563 (EAS)	Our cohort
c.592+1G>A	NA	1	0.00005563 (EAS)	Our cohort
*POU3F4*				
c.346dupG	p.A116Gfs *77	2	No data	Our cohort
c.919_921delGAG	p.E307del	1	No data	Our cohort
c.950T>A	p.L317 *	1	No data	Our cohort
c.1084T>C	p.*362Rext *113	1	No data	Our cohort
*EYA1*				
c.1081C>T	p.R361 *	2	No data	Our cohort; Ref [36]
c.1540_1542delCTG	p.L514del	2	No data	Our cohort
c.385C>T	p.Q129 *	1	No data	Our cohort
c.403G>A	p.G135S	1	0.0007082 (EAS)	Our cohort
c.466C>T	p.Q156 *	1	No data	Our cohort; Ref [44]
c.1360+5G>A	NA	1	No data	Our cohort; Ref [36]
c.1735delG	p.D579Tfs *60	1	No data	Our cohort; Ref [44]
*TECTA*				
c.5372C>G	p.P1791R	1	0.001035 (EAS)	Our cohort
c.5471G>A	p.G1824D	1	0.0005513 (EAS)	Our cohort
c.6062G>A	p.R2021H	1	0.000008791 (EUP-NF)	Our cohort
*POU4F3*				
c.491C>G	p.P164R	1	0.0004023 (EAS)	Our cohort
c.982A>G	p.K328E	1	No data	Our cohort; Ref [35]
*MITF*				
c.862dupA	p.I288Nfs *9	1	No data	Our cohort
c.1078C>T	p.R360 *	1	No data	Our cohort
c.938-1G>A	NA	1	No data	Our cohort
*PJVK*				
c.406C>T	p.R136 *	1	0.00008316 (AFR)	Our cohort
c.593C>A	p.A198D	1	0.0005119 (EAS)	Our cohort
*COL4A5*				
c.367delG	p.G123Dfs *32	1	No data	Our cohort
c.796C>T	p.R266 *	1	No data	Our cohort
*WFS1*				
c.2051C>T	p.A684V	2	No data	Our cohort
*SIX5*				
c.1872dupC	p.A625Rfs *15	1	No data	Our cohort
*GATA3*				
c.149delT	p.F51Lfs *144	1	No data	Our cohort; Ref [34]
c.477delG	p.D160Tfs *35	1	No data	Our cohort
*TMPRSS3*				
c.916G>A	p.A306T	2	0.0006014 (EAS)	Our cohort
c.432delA	p.Q144Hfs *8	1	0.0005012 (EAS)	Our cohort
c.743C>T	p.T248M	1	0.0004894 (EAS)	Our cohort
*PAX3*				
c.52C>T	p.Q18 *	1	No data	Our cohort
c.587-2A>G	NA	1	0.000109 (EAS)	Our cohort
*USH2A*				
c.1614C>A	p.C538*	1	No data	Our cohort
c.4576G>A	p.G1526R	1	0.0002521 (EAS)	Our cohort
c.7045dupT	p.W2349Lfs *8	1	No data	Our cohort
c.8559-2A>G	NA	1	0.0004353 (EAS)	Our cohort
*MYO6*				
c.3736delC	p.P1246Qfs *39	1	No data	Our cohort
c.1675-2A>C	NA	1	No data	Our cohort
*OTOG*				
c.3582C>A	p.Y1194 *	1	0.0009720 (EAS)	Our cohort
c.4023_4045del	p.Q1342Pfs *104	1	No data	Our cohort
*ATP6V1B2*				
c.1516C>T	p.R506 *	1	No data	Our cohort
*SOX10*				
c.314_315del	p.K105Tfs *28	1	No data	Our cohort
*PTPRQ*				
c.6087-3T>G	NA	3	0.002970 (EAS)	Our cohort
c.3181delC	p.L1061Ffs *11	1	No data	Our cohort
*EPS8L2*				
c.1304G>A	p.W435 *	2	0.0001784 (EAS)	Our cohort
*EDNRB*				
c.754-2A>G	NA	1	No data	Our cohort

All NCBI Reference Sequences are listed in Supplementary Material Table S1. del: deletion; dup: duplication; fs: frameshift; *: termination; ext: extension. EAS: East Asian; AFR: African; EUP-NF: European-non-Finnish.

## Supplementary Materials

The following are available online at https://www.mdpi.com/2073-4425/10/10/772/s1, Table S1: All NCBI Reference Sequences of other deafness genes.
