# Supplementary material for: Genetic Epidemiology and Clinical Features of Hereditary Hearing Impairment in the Taiwanese Population
Source: Genes (Basel). 2019 Oct 1;10(10):772. doi: 10.3390/genes10100772 (PMC6826657; doi:10.3390/genes10100772)
Supplement: Supplementary file 1 [file genes-10-00772-s001.pdf]

Supplementary Material

Table S1. All NCBI Reference Sequences of other deafness genes.

Variants	NCBI Reference Sequence
KCNQ4	NM_004700.3 / NP_004691.2
MYO7A	NM_000260.3 / NP_000251.3
POU3F4	NM_000307.4 / NP_000298.3
EYA1	NM_000503.5 / NP_000494.2
TECTA	NM_005422.2 / NP_005413.2
POU4F3	NM_002700.3 / NP_002691.1
MITF	NM_198159.2 / NP_937802.1
PJVK	NM_001042702.4 / NP_001036167.1
COL4A5	NM_033380.2 / NP_203699.1
WFS1	NM_006005.3 / NP_005996.2
SIX5	NM_175875.5 / NP_787071.3
GATA3	NM_001002295.2 / NP_001002295.1
TMPRSS3	NM_024022.3 / NP_076927.1
PAX3	NM_181459.4 / NP_852124.1
USH2A	NM_206933.3 / NP_996816.2
MYO6	NM_004999.4 / NP_004990.3
OTOG	NM_001277269.1 / NP_001264198.1
ATP6V1B2	NM_001693.4 / NP_001684.2
SOX10	NM_006941.3 / NP_008872.1
PTPRQ	NM_001145026.2 / NP_001138498.1
EPS8L2	NM_022772.4 / NP_073609.2
EDNRB	NM_001201397.1 / NP_001188326.1
